# Fucoidan Modulates Osteoarthritis Progression Through miR-22/HO-1 Pathway

**DOI:** 10.3390/cells14151208

**Published:** 2025-08-06

**Authors:** Tsung-Hsun Hsieh, Jar-Yi Ho, Chih-Chien Wang, Feng-Cheng Liu, Chian-Her Lee, Herng-Sheng Lee, Yi-Jen Peng

**Affiliations:** 1 Graduate Institute of Medical Sciences, National Defense Medical Center, Taipei 114, Taiwan; jason9313@hotmail.com; 2 Department of Orthopedics and Traumatology, Taipei Veterans General Hospital, Taipei 112, Taiwan; 3 Graduate Institute of Life Sciences, National Defense Medical Center, Taipe 114, Taiwan; jaryiho@gmail.com; 4 Department of Orthopedics, Tri-Service General Hospital, National Defense Medical Center, Taipei 114, Taiwan; tsghcc@gmail.com; 5 Rheumatology/Immunology and Allergy, Department of Medicine, Tri-Service General Hospital, National Defense Medical Center, Taipei 114, Taiwan; lfc10399@yahoo.com.tw; 6 Department of Orthopedics, School of Medicine, College of Medicine, Taipei Medical University Hospital, Taipei 114, Taiwan; chianherlee@yahoo.com.tw; 7 Department of Pathology and Laboratory Medicine, Kaohsiung Veterans General Hospital, Kaohsiung 813, Taiwan; herngsheng131419@gmail.com; 8 Department of Pathology, Tri-Service General Hospital, National Defense Medical Center, Taipei 114, Taiwan

**Keywords:** osteoarthritis, miR-22/HO-1 pathway, fucoidan, chondrocytes, synovial fibroblasts

## Abstract

Introduction: Osteoarthritis (OA), a leading cause of disability among the elderly, is characterized by progressive joint tissue destruction. Fucoidan, a sulfated polysaccharide with known anti-inflammatory and antioxidant properties, has been investigated for its potential to protect against interleukin-1 beta (IL-1β)-induced articular tissue damage. Methods: Human primary chondrocytes and synovial fibroblasts were pre-treated with 100 μg/mL fucoidan before stimulation with 1 ng/mL of IL-1β. The protective effects of fucoidan were assessed by measuring oxidative stress markers and catabolic enzyme levels. These in vitro findings were corroborated using a rat anterior cruciate ligament transection-induced OA model. To explore the underlying mechanisms, particularly the interaction between microRNAs (miRs) and heme oxygenase-1 (HO-1), five candidate miRs were identified in silico and experimentally validated. Luciferase reporter assays were used to confirm direct interactions. Results: Fucoidan exhibited protective effects against IL-1β-induced oxidative stress and catabolic processes in both chondrocytes and synovial fibroblasts, consistent with in vivo observations. Fucoidan treatment restored HO-1 expression while reducing inducible nitric oxide synthase and matrix metalloproteinase levels in IL-1β-stimulated cells. Notably, this study revealed that fucoidan modulates the miR-22/HO-1 pathway, a previously uncharacterized mechanism in OA. Specifically, miR-22 was upregulated by IL-1β and subsequently attenuated by fucoidan. Luciferase reporter assays confirmed a direct interaction between miR-22 and HO-1. Conclusion: The results demonstrate that fucoidan mitigates OA-related oxidative stress in chondrocytes and synovial fibroblasts through the novel modulation of the miR-22/HO-1 axis. The miR-22/HO-1 pathway represents a crucial therapeutic target for OA, and fucoidan may offer a promising therapeutic intervention.

## 1. Introduction

Osteoarthritis (OA), the most prevalent degenerative joint disease, is caused by multiple risk factors, including aging, obesity, and joint injury. These factors ultimately lead to osteophyte formation, cartilage degradation, and synovitis, significantly impairing quality of life, particularly in elderly individuals [1,2,3]. The pathogenesis of OA is characterized by an imbalance of extracellular matrix (ECM) catabolism and anabolism in chondrocytes, driven by the activation of cytokines and increasing oxidative stress [4]. Interleukin-1β (IL-1β) is crucial in the process, stimulating the production of inducible nitric oxide synthase (iNOS) and matrix metalloproteinases (MMPs) [5,6]. The resulting nitric oxide (NO) and reactive oxygen species (ROS) interact with superoxide anion, exacerbating oxidative stress and contributing to cellular dysfunction and cartilage degeneration [7,8]. Additionally, IL-1β activates the mitogen-activated protein kinase (MAPK) and nuclear factor-kappa B (NF-κB) signaling pathways, further disrupting cellular homeostasis and promoting catabolic effects [9,10]. In contrast, nuclear factor-erythroid 2-related factor2 (Nrf2), a master regulator of redox balance, mitigates oxidative stress by upregulating heme oxygenase 1 (HO-1) [11,12]. Moreover, Nrf2 inhibits NF-κB activation, offering potential protection against cartilage damage during OA progression [13,14]. Therefore, suppressing IL-1β-mediated catabolic pathways and simultaneously activating the Nrf2/HO-1 signaling axis represent promising therapeutic strategies for OA.

MicroRNAs (miRNAs) have emerged as pivotal regulators in the complex pathogenesis of osteoarthritis, influencing various cellular processes that contribute to disease progression [15]. These small non-coding RNAs modulate gene expression at the post-transcriptional level, impacting pathways crucial for cartilage homeostasis and inflammation [16]. For example, miR-140 plays a protective role by maintaining the chondrocyte phenotype and suppressing catabolic gene expression, while the dysregulation of this miR-140 is associated with increased cartilage degradation [17]. Conversely, miRNAs such as miR-21 promote cartilage degradation by targeting tissue inhibitors of metalloproteinase-3 (TIMP3), thereby facilitating MMP activity [18]. Furthermore, miRs are deeply involved in the inflammatory process of OA, with some miRNAs regulating the production of inflammatory cytokines via pathways such as NF-κB, which plays a pivotal role in osteoarthritic synovial inflammation [19]. Interestingly, hydroxytryrosol, a nutraceutical compound, has demonstrated chondroprotective effects by regulating the miR-9/sirtuin-1 axis [20]. However, the precise regulatory networks of miR interactions in the joint environment, as well as how subtle variations in their expression patterns contribute to the heterogeneity and progression of OA, require further exploration using systems biology approaches.

Considering the limitations and side effects of current OA treatments, this study aimed to evaluate the therapeutic potential of fucoidan, a natural sulfated polysaccharide extracted from brown seaweed [21]. Previous studies have indicated that fucoidan can alleviate IL-1β-induced inflammation in chondrocytes and monosodium iodoacetate-induced osteoarthritis in animal models [22,23]. Building on this, we focused on elucidating the mechanisms by which fucoidan inhibits oxidative stress and modulates microRNA signaling pathways in articular chondrocytes, synovial fibroblasts, and traumatic osteoarthritic animal models. The goal of this study was to assess how fucoidan modulates key mechanisms relevant to therapeutic interventions in OA.

## 2. Materials and Methods

### 2.1. Reagents

The fucoidan powder used in this study was sourced from the commercial product Hi-Q Oligo-fucoidan, manufactured by Hi-Q Marine Biotech International Ltd. in New Taipei City, Taiwan. This product contains fucoidan extracted from brown seaweed through enzyme hydrolysis and filtration using molecular weight cut-off membranes to achieve molecular weights within the range of 500 to 1500 Da, with an average molecular weight of 800 Da [21,24]. The fucoidan powder was dissolved in deionized water (ddH_2_O) and stored at −20 °C until use. The IL-1β used in this study was purchased from R&D Systems. Antibodies for Western blotting were obtained from several sources: anti-iNOS, MMP-1, MMP-3, HO-1, and Nrf2 from Abcam (Cambridge, MA, USA); the internal control anti-β-actin from Santa Cruz Biotechnology (Dallas, TX, USA); and signal transduction antibodies for key pathways (NF-κB, ERK1/2, SAPK/JNK, and p38, including both phospho- and total forms) from Cell Signaling Technology (Danvers, MA, USA). Secondary antibodies (anti-rabbit IgG HRP and anti-mouse IgG HRP) were procured from Agilent Technologies DAKO (Santa Clara, CA, USA).

### 2.2. Source and Culture of Human Joint Cells: Articular Chondrocytes and Synovial Fibroblasts

Human articular chondrocytes and synovial fibroblasts were isolated from OA patients undergoing total knee replacement, following informed consent and ethical approval from the Tri-Service General Hospital Institutional Review Board (TSGHIRB no.: 1-102-05-091; case number: 20). For chondrocyte isolation, cartilage fragments were treated with 0.25% trypsin (Gibco, Carlsbad, CA, USA) for 30 min and then digested with collagenase type H and type Ⅱ (Sigma-Aldrich, Merck KGaA, Darmstadt, Germany) for 16–18 h at 37 °C in 5% CO_2_. After filtration (40 μm strainer, Merck Millipore, Billerica, MA, USA) and washing, the chondrocytes were cultured in DMEM/F-12 (Gibco) with 10% FBS (Gibco) and antibiotics. Synovial fibroblasts were obtained by digesting minced synovial tissues with collagenase type I (Gibco) in serum-free F-12 HAM medium (Gibco) for 18–24 h, followed by filtration (70 μm strainer, Millipore) and culture in F-12 HAM medium supplemented with 10% FBS (Sigma-Aldrich, St. Louis, MO, USA) and antibiotics. Both cell types were used between passages 3 and 5 after culturing in a humidified 5% CO_2_ incubator at 37 °C. Primary cell experiments were conducted using cells from at least three independent human donors. To account for inter-donor variability, data were analyzed using a linear mixed-effects model, with donor specified as a random effect. Where appropriate, sensitivity analyses were performed by systematically excluding each donor to assess the robustness of the observed effects.

### 2.3. Cell Viability Assays

The viability of the human chondrocytes and synovial fibroblasts was determined using an MTT assay. The cells (1 × 10^4^ per well) were seeded in 96-well plates (0.2 mL medium) and incubated with fucoidan at concentrations ranging from 0 to 200 μg/mL (0, 5, 10, 50, 100, and 200 μg/mL) for 24 and 48 h in triplicate. Following treatment, MTT solution (50 μL/well) was added, and the plates were incubated for 3 h at 37 °C. The resulting formazan was solubilized with 150 μL of dimethyl sulfoxide under shaking for 10 min. Absorbance at 562 nm was then measured using a microplate reader from Bio-Tek Instruments Inc. (Winooski, VT, USA).

### 2.4. Protein Extraction and Western Blotting

For protein analysis, cells were stimulated as indicated, washed with ice-cold PBS, and lysed using RIPA buffer (Sigma) containing 100 μM Na_3_VO_4_ and a protease inhibitor cocktail (Sigma) at 4 °C for 15 min. Protein concentrations were measured using a BCA Protein Assay Kit (Thermo Pierce, Thermo Fisher Scientific Inc., Waltham, MA, USA). Equal amounts of protein (20 μg) were then separated via SDS-PAGE and transferred to PVDF membranes. Following a 1-h block with 2% BSA at 26 °C, membranes were probed overnight at 4 °C with primary antibodies diluted in TBST. After washing, the membranes were incubated for 1 h at room temperature with HRP-labeled secondary antibodies (1:5000, Agilent Technologies DAKO, Santa Clara, CA, USA). Protein visualization was achieved using Western HRP Substrate, and immunoblot images were captured using a UVP BioSpectrum AC image system (UVP, Upland, CA, USA).

### 2.5. Measurement of Nitrite Production in Culture Medium

Cells (1 × 10^4^ per well) were incubated in 24-well plates overnight. The culture supernatant was collected, and NO production was measured using the Giess reaction to determine the nitrite concentrations with a spectrophotometer. The supernatant (100 μL) was incubated with 50 μL of 0.1% sulfanilamide in 5% phosphoric acid and 50 μL of 0.1% N-1-naphthylethylenediamine dihydrochloride. After a 30-min incubation at room temperature, absorbance was measured at 550 nm using a microplate reader (Bio-Tek Instruments Inc., Winooski, VT, USA).

### 2.6. Measurement of ROS

To assess intracellular reactive oxygen species (ROS) levels, human chondrocytes and synovial fibroblasts (1 × 10^4^ per well) were seeded and incubated overnight at 37 °C in a 5% CO_2_ atmosphere. After treatment with fucoidan or IL-1β, the cells were loaded with 20 μM DCFH-DA (Sigma) and incubated for 30 min at 37 °C. Subsequently, the cells were washed with PBS, and fluorescence intensity was visualized using an Olympus microscope (Tokyo, Japan).

### 2.7. Animals and Experimental Design

In this study, we used 8-week-old male Sprague–Dawley rats (200–250 g) obtained from Bio-LASCO (Yi-Lan, Taiwan). All animal experiments were performed with the approval of the National Defense Medical Center Institutional Animal Care and Use Committee (IACUC-16-267) and in accordance with NIH guidelines. An a priori power analysis was conducted using the resource equation method. With 3 experimental groups and 9 animals per group, the total number of animals was 27, yielding an E value of 24. Although this slightly exceeds the generally recommended range of 10 to 20, the design was considered appropriate in light of the anticipated biological variability and need to ensure sufficient statistical power [25]. Using a computer-generated random number sequence, the 27 rats were randomly assigned to three groups (*n* = 9 per group): a sham surgery group and two anterior cruciate ligament (ACLT) surgery groups (right knee). Allocation concealment was maintained by assigning the animals through sealed envelopes. One week following surgery, one of the ACLT groups received daily oral fucoidan (100 mg/kg), while the other received a vehicle. After thirteen weeks, knee joints were harvested, fixed in 10% formalin, decalcified, and processed for paraffin embedding. Joint damage was assessed in 4 μm thick sections stained with H&E and Safranin O, using a light microscope and the OARSI scoring system (0–6.5, mild to severe) [26]. Histological evaluations were performed by an investigator blinded to the group assignments.

### 2.8. RNA Extraction and Real-Time PCR Analysis

Total RNA was isolated using an miRNeasy kit (QIAGEN, Germantown, MD, USA) following the manufacturer’s protocol. Subsequently, cDNA was synthesized from equal amounts of RNA using a miScript II RT kit (QIAGEN). Real-time PCR was performed using a miScript SYBR Green PCR kit (QIAGEN) on an ABI QuantStudio-5 Instrument (Bedford, MA, USA). The PCR conditions were as follows: 95 °C for 15 min, 40 cycles of 94 °C for 15 s, 55 °C for 30 s, and 70 °C for 30 s. U6 RNA was used as an internal control to normalize miR-22 expression, and the 2^−ΔΔCq^ method was used to calculate the fold changes in expression.

### 2.9. Luciferase Reporter Assay

Reporter constructs, pMIR-HO-1-WT and pMIR-HO-1-MUT, containing the wild-type and mutant miR-22 binding sites of the HMOX1 3′-UTR, respectively, were generated by cloning PCR products into the pMIR-reporter vector (Invitrogen, Waltham, MA, USA) between the HindIII and SpeI sites. These constructs (50 ng) were co-transfected into articular chondrocytes and synovial fibroblasts, along with either 100 nM miR-22 mimics or negative control mimics (NC) using DharmaFECT transfection reagent (Horizon Discovery Biosciences Ltd., Lafayette, CO, USA). To normalize transfection efficiency, a β-gal control vector (500 ng) was co-transfected in all experiments. Luciferase and β-galactosidase activities were measured 48 h post-transfection using the Dual-Light Luciferase and β-Galactosidase Reporter Gene Assay System (Invitrogen, Waltham, MA, USA), as described by the manufacturer. Luminescence was detected using a Synergy HT Multi-Mode Microplate Reader (BioTek Instruments, Winooski, VT, USA), with four replicates for each experimental group.

### 2.10. Statistical Analysis

A statistical analysis was performed on data obtained from at least three independent experiments. The results are presented as mean ± standard deviation (SD). The expression levels of target proteins, normalized to the internal control, are shown as fold changes. For comparisons between multiple groups, a one-way ANOVA was used, followed by Dunnett’s post hoc test to address potential non-normality of the data. A significance level of *p* < 0.05 was adopted for all tests. Data processing and analysis were carried out using ImageJ (latest v. 1.54p, NIH, Bethesda, MD, USA) and GraphPad Prism 5.0 software (GraphPad Software, San Diego, CA, USA).

## 3. Results

### 3.1. Effects of Fucoidan on Cell Viability in Articular Chondrocytes and Synovial Fibroblasts

**Figure 1:**
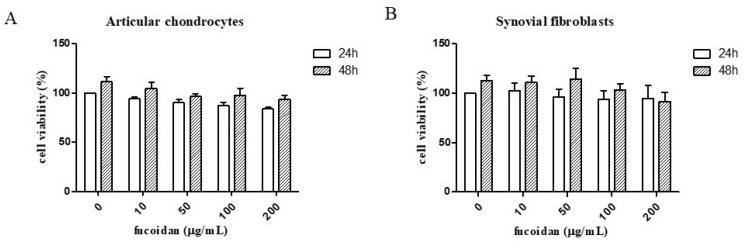

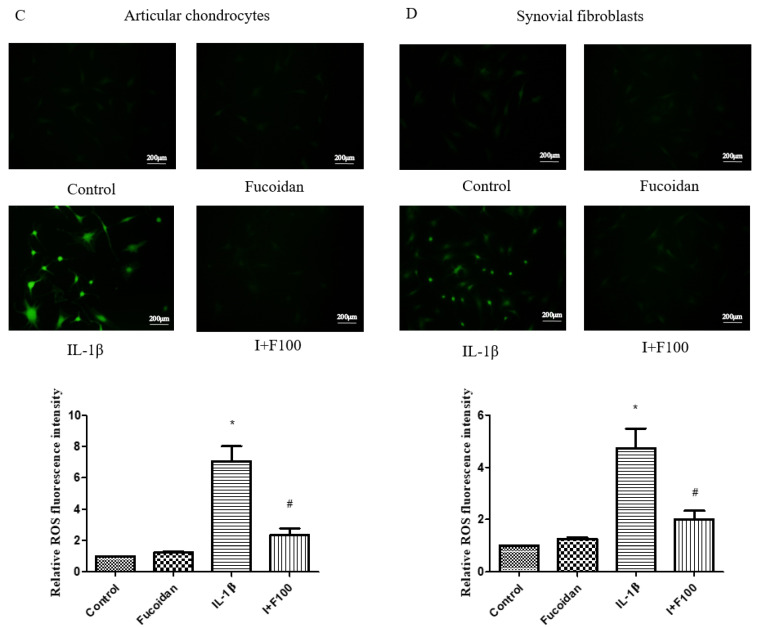
The effects of fucoidan in the MTT assay and on IL-1β-induced reactive oxygen species (ROS). The cell viability of (**A**) articular chondrocytes and (**B**) synovial fibroblasts after fucoidan treatment alone examined in a dose-dependent manner (0, 10, 50,100, 200 μg/mL) for 24 or 48 h (*n* = 6). The ROS expression of (**C**) articular chondrocytes and (**D**) synovial fibroblasts. The high level of ROS induced by IL-1β was suppressed by fucoidan treatment in articular chondrocytes and synovial fibroblasts (* *p* < 0.05 compared with control; # *p* < 0.05, IL-1β vs. I+F100).

The MTT assay results showed that fucoidan did not significantly affect cell survival at concentrations from 0 to 200 μg/mL in either primary human articular chondrocytes (Figure 1A) or synovial fibroblasts (Figure 1B).

### 3.2. Fucoidan Suppresses IL-1β-Induced Reactive Oxygen Species and Catabolic Factors in Articular Chondrocytes and Synovial Fibroblasts

The results show that stimulation with 1 ng/mL of IL-1β significantly increased ROS production by 5.26 ± 0.57-fold (*n* = 4, *p* < 0.01) in articular chondrocytes (Figure 1C) and by 4.98 ± 0.91-fold (*n* = 4, *p* < 0.05) in synovial fibroblasts (Figure 1D) compared to the control group. However, co-incubation with fucoidan markedly suppressed IL-1β-induced ROS generation in a dose-dependent manner.

To further investigate the impact of fucoidan on oxidative stress-related molecules, we performed a Western blot analysis. Treatment with 1 ng/mL IL-1β for 24 h significantly increased inducible nitric oxide synthase (iNOS) protein expression by 5.26 ± 0.57-fold (*n* = 4, *p* < 0.01) in articular chondrocytes and by 4.98 ± 0.91-fold (*n* = 4, *p* < 0.05) in synovial fibroblasts compared to the control. Fucoidan treatment alone (100 μg/mL) did not significantly affect iNOS expression; however, co-treatment with fucoidan attenuated IL-1β-induced iNOS expression in a dose-dependent manner.

**Figure 2:**
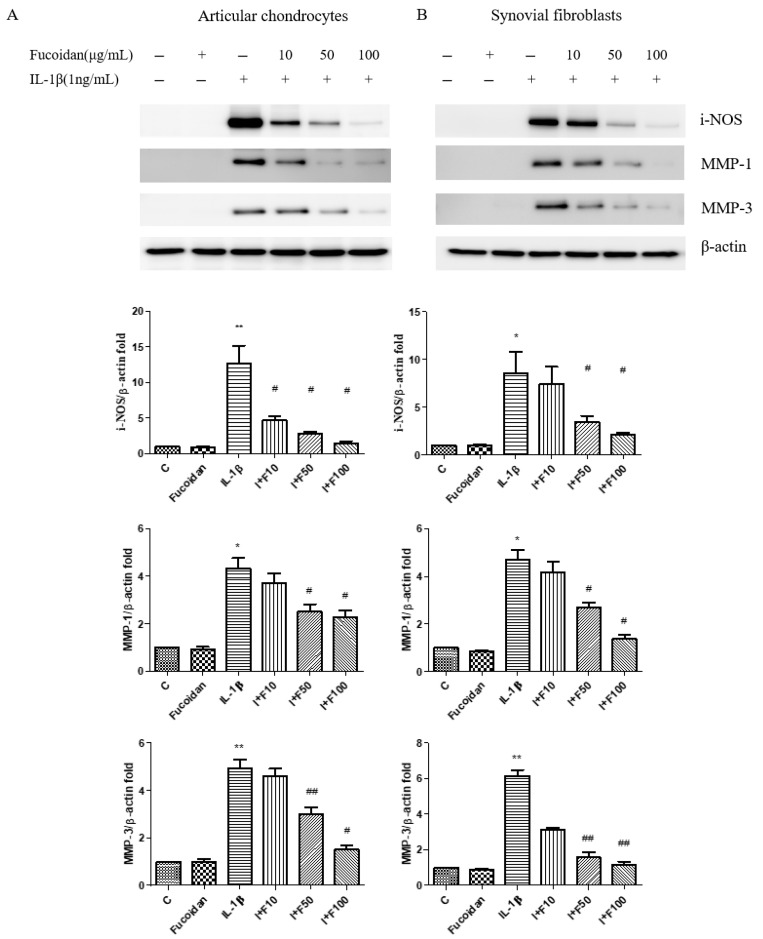

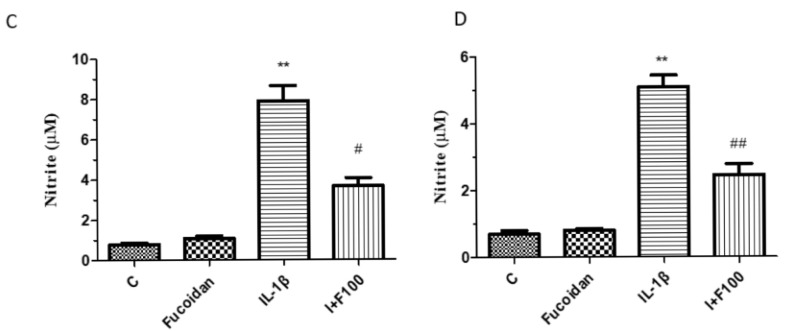
The effects of fucoidan on IL-1β-induced i-NOS, MMP-1, and MMP-3 protein expression and NO production in articular chondrocytes and synovial fibroblasts. (**A**,**B**) Upregulation of i-NOS, MMP-1, and MMP-3 induced by IL-1β was suppressed by fucoidan treatment in articular chondrocytes and synovial fibroblasts. (**C**,**D**) Griess reaction. Upregulation of nitrite production induced by IL-1β was suppressed by fucoidan treatment in articular chondrocytes and synovial fibroblasts (* *p* < 0.05 compared with control; ** *p* < 0.01 compared with control; # *p* < 0.05, IL-1β vs. I+F100; ## *p* < 0.01, IL-1β vs. I+F100).

Similarly, IL-1β stimulation significantly increased the expression of matrix metalloproteinases (MMP-1 and MMP-3) in both cell types. In articular chondrocytes, the MMP-1 and MMP-3 expression levels increased by 4.33 ± 0.76-fold (*n* = 4, *p* < 0.05) and 4.93 ± 0.64-fold (*n* = 4, *p* < 0.01), respectively (Figure 2A). In synovial fibroblasts, they increased by 4.69 ± 0.75-fold (*n* = 4, *p* < 0.05) and 6.15 ± 0.57-fold (*n* = 4, *p* < 0.01), respectively (Figure 2B). These effects were significantly suppressed by co-incubation with fucoidan in a dose-dependent manner. Additionally, the culture medium was collected to evaluate nitrite production. IL-1β treatment significantly increased NO production to 7.94 ± 1.51 μM (*n* = 4, *p* < 0.01) in articular chondrocytes (Figure 2C) and to 5.08 ± 0.71 μM (*n* = 4, *p* < 0.01) in synovial fibroblasts (Figure 2D). Co-treatment with fucoidan effectively suppressed IL-1β-induced nitrite production in a dose-dependent manner.

### 3.3. Effects of Fucoidan on IL-1β-Induced NF-κB Activation and MAPK Signaling in Articular Chondrocytes and Synovial Fibroblasts

**Figure 3:**
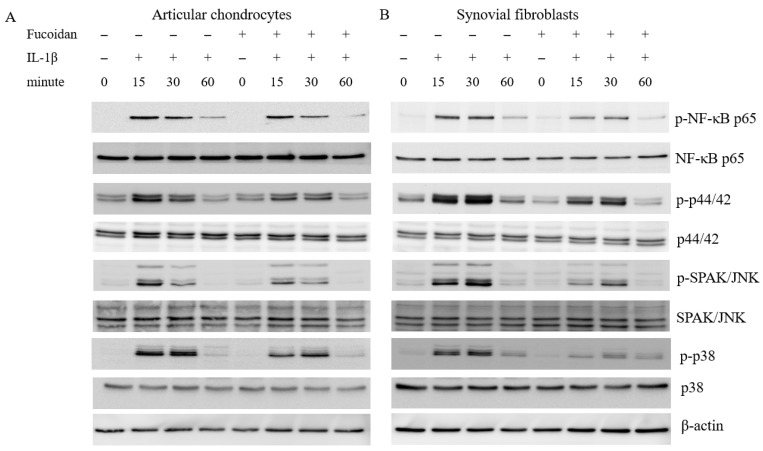

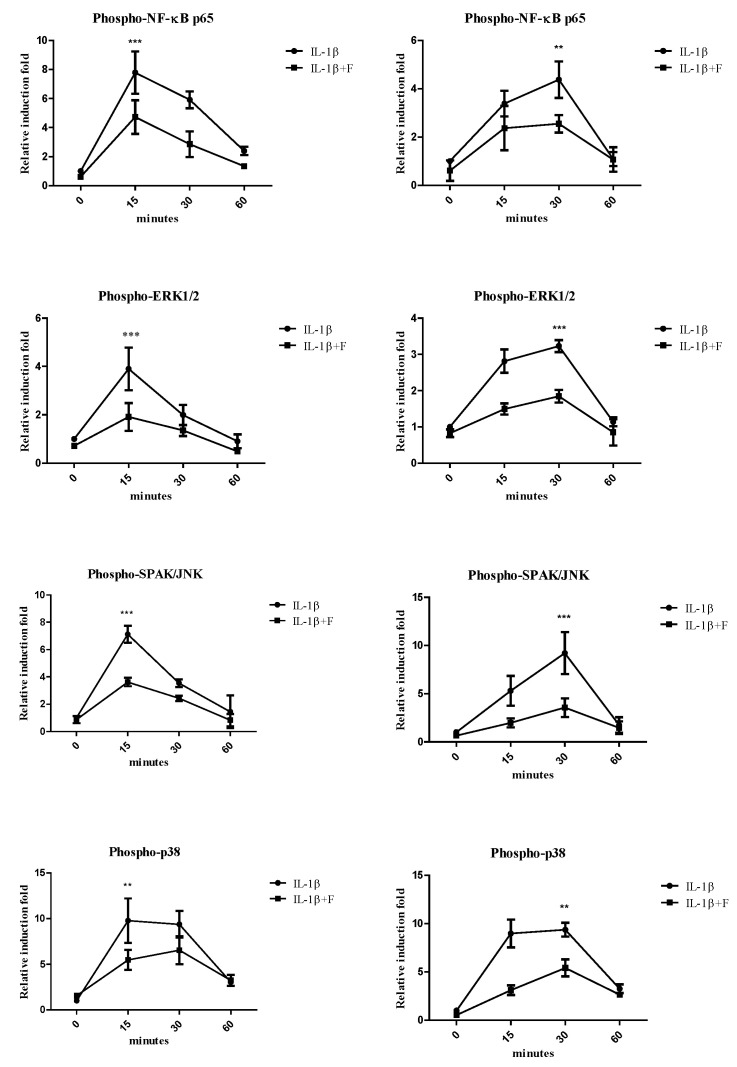
The effects of fucoidan on IL-1β-induced NF-κB activation and MAP kinases in (**A**) articular chondrocytes and (**B**) synovial fibroblasts (** *p* < 0.01 compared with the same minutes, IL-1β vs. I+F; *** *p* < 0.001, compared with the same minutes, IL-1β vs. I+F).

To explore the signaling pathways involved in the antioxidative effects of fucoidan on articular chondrocytes and synovial fibroblasts, we examined the activation of ERK1/2, SAPK/JNK, p38 MAP kinases, and NF-κB. Stimulation with IL-1β induced the phosphorylation of ERK1/2, SAPK/JNK, p38 MAP kinases, and NF-κB p65 for 15, 30, and 60 min in both cells. In articular chondrocytes (Figure 3A), the phosphorylation of ERK1/2, SAPK/JNK, p38, and NF-κB p65 peaked 15 min following IL-1β stimulation. In synovial fibroblasts (Figure 3B), the phosphorylation of these proteins reached its peak 30 min after IL-1β stimulation. Notably, co-incubation with fucoidan (100 μg/mL) effectively suppressed the IL-1β-induced phosphorylation of ERK1/2, SAPK/JNK, p38 MAP kinases, and NF-κB p65 in both articular chondrocytes and synovial fibroblasts.

### 3.4. Fucoidan Activated the Nrf2/HO-1 Pathway in Articular Chondrocytes and Synovial Fibroblasts

**Figure 4:**
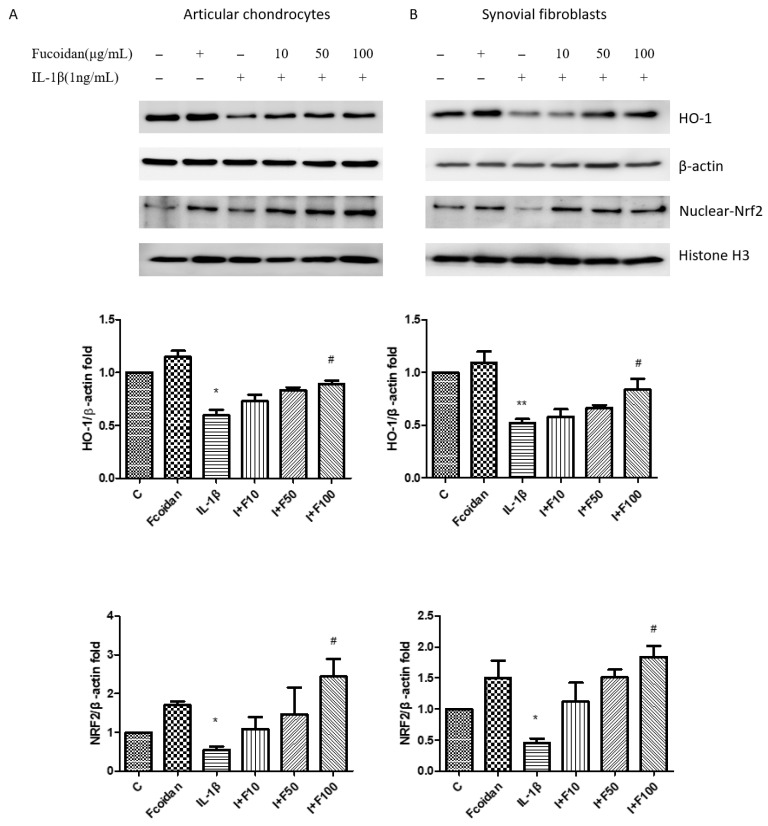
The effects of fucoidan on the IL-1β-induced HO-1/Nrf2 signaling pathway in (**A**) articular chondrocytes and (**B**) synovial fibroblasts. The downregulation of HO-1 and Nrf2 induced by IL-1β was increased by fucoidan treatment in articular chondrocytes and synovial fibroblasts (* *p* < 0.05 compared with control; ** *p* < 0.01 compared with control; # *p* < 0.05, IL-1β vs. I+F100).

Previous studies have demonstrated that fucoidan can reduce oxidative stress by upregulating HO-1 protein expression [27,28,29]. The effects of fucoidan on articular chondrocytes and synovial fibroblasts remain unclear. Therefore, we investigated the involvement of the Nrf2/HO-1 signaling pathway in the response to fucoidan treatment. A Western blot analysis showed that incubation with 1 ng/mL IL-1β for 24 h significantly decreased HO-1 protein expression to 0.59 ± 0.09-fold (*n* = 4, *p* < 0.05) in articular chondrocytes (Figure 4A) and to 0.52 ± 0.06-fold (*n* = 4, *p* < 0.01) in synovial fibroblasts (Figure 4B) compared to the control group. Treatment with fucoidan alone (100 μg/mL) did not significantly affect HO-1 expression; however, co-incubation with fucoidan effectively reversed the IL-1β-induced downregulation of HO-1 protein expression in a dose-dependent manner. Furthermore, a Western blot analysis of nuclear and cytoplasmic fractions revealed that fucoidan promoted Nrf2 accumulation and translocation into the nucleus in both articular chondrocytes and synovial fibroblasts, suggesting activation of the Nrf2/HO-1 signaling pathway.

### 3.5. Inverse Regulation of miR-22 and HO-1 Expression in Response to IL-1β and Fucoidan in Chondrocytes and Synovial Fibroblasts

**Figure 5:**
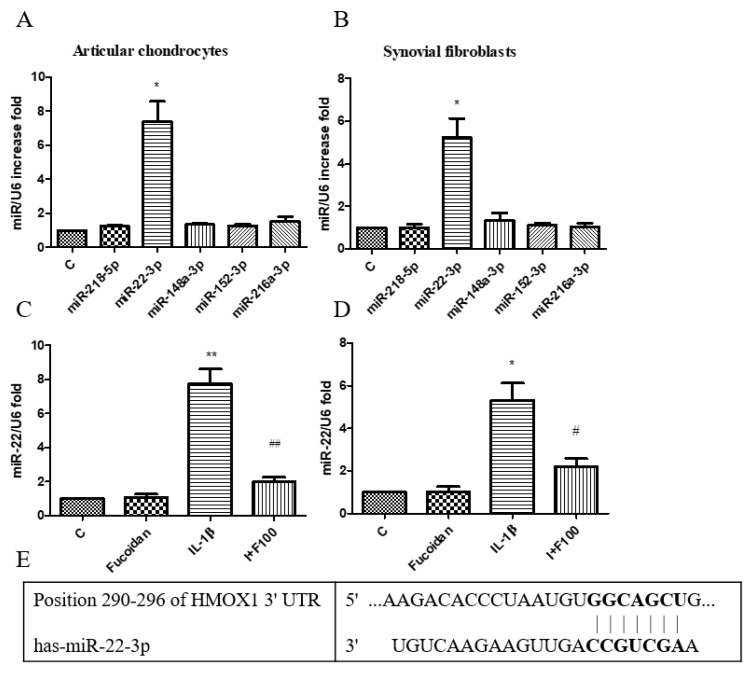
Effects of fucoidan on IL-1β-induced miR regulation in articular chondrocytes and synovial fibroblasts. (**A**,**B**) Total RNA was isolated from articular chondrocytes and synovial fibroblasts treated with IL-1β for 24 h. Real-time PCR analysis of miR-218-5p, miR-22-3p, miR-148a-3p, miR-152-3p, and miR-216a-3p expression (*n* = 5). (**C**,**D**) Fucoidan attenuates IL-1β-induced oxidative stress via downregulation of miR-22. Real-time PCR analysis of miR-22 in articular chondrocytes and synovial fibroblasts incubated with fucoidan in response to IL-1β (* *p* < 0.05 compared with control; ** *p* < 0.01 compared with control; # *p* < 0.05, IL-1β vs. I+F100; ## *p* < 0.01, IL-1β vs. I+F100). (**E**) Site of matching between HMOX-1 3′UTR and miR-22 sequences (provided by TargetScan database).

According to our previous findings, incubation with 1 ng/mL of IL-1β for 24 h significantly reduced HO-1 protein expression in both articular chondrocytes and synovial fibroblasts. However, co-incubation with fucoidan effectively restored HO-1 expression (Figure 4). As microRNAs are critical in osteoarthritis progression, we investigated whether specific miRNAs regulate HO-1 expression. Using the TargetScan and miRBase databases, we predicted potential miRNA candidates targeting HO-1. Five miRNAs with the highest probability of conserved targeting (P_CT_) scores were identified: miR-218-5p, miR-22-3p, miR-148a-3p, miR-152-3p, and miR-216a-3p. Among these, no significant changes were observed in the expression levels of miR-218-5p, miR-148a-3p, miR-152-3p, or miR-216a-3p following IL-1β stimulation. However, miR-22 expression was significantly altered in response to IL-1β treatment in both articular chondrocytes (Figure 5A) and synovial fibroblasts (Figure 5B). To further confirm this, we measured mature miR-22 expression using specific primers capable of distinguishing mature miRNAs from their precursors. The results demonstrated that IL-1β stimulation significantly increased miR-22 levels after 24 h, while co-incubation with fucoidan markedly suppressed this IL-1β-induced upregulation in both articular chondrocytes (Figure 5C) and synovial fibroblasts (Figure 5D). Furthermore, a TargetScan analysis predicted a specific binding site for miR-22 within the 3′-UTR of HMOX-1 (HO-1) mRNA, as shown in Figure 5E. The predicted binding site involves a 7-mer seed match, indicating that miR-22 may directly regulate HO-1 expression by binding to its complementary sequence.

### 3.6. Direct Target of HMOX1 3′-UTR by miR-22 in Chondrocytes and Synovial Fibroblasts

**Figure 6:**
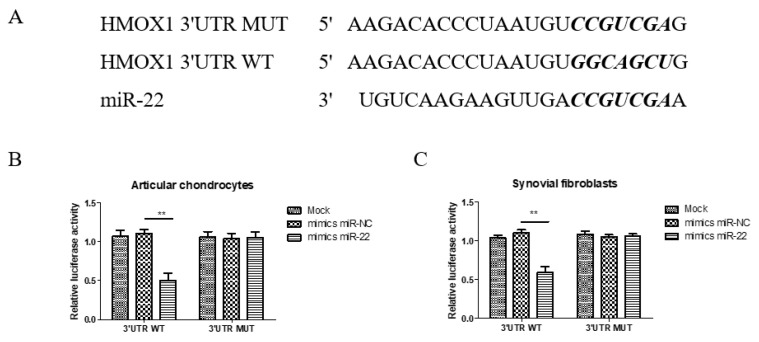
The HMOX1 3′-UTR is a direct target of miR-22 in articular chondrocytes and synovial fibroblasts. (**A**) The predicted miR-22 targeting site within the HO-1 3′UTR and the mutated sites aligned as indicated. (**B**,**C**) The relatives of dual luciferase activity in articular chondrocytes and synovial fibroblasts were determined after the empty pMIR reporter (Mock), wild-type 3′UTR, or mutant 3′UTR plasmids were co-transfected with the negative control or miR-22 (** *p* < 0.01, miR-NC vs. miR-22).

As identified in the TargetScan database, the typical miR-22 binding seed sequence aligned in the HMOX1-3′-UTR (Figure 6A). To confirm the specificity of the miR-22 binding seed sequences, the miR-22 inhibitory capacity was evaluated with luciferases fused with a wild-type fragment of HMOX1 (pMIR-HO-1-3′UTR-WT) or a mutant fragment of the seed sequences (pMIR-HO-1-3′UTR-MUT) in articular chondrocytes and synovial fibroblasts. In luciferase assays, miR-22 mimics only significantly reduced the activity of the luciferase carrying the wild-type HMOX1 3′-UTR but lost its inhibitory ability in the luciferase carrying the mutant HMOX1 3′-UTR, with no difference compared with the NC controls in either articular chondrocytes (Figure 6B) or synovial fibroblasts (Figure 6C). These results indicate that miR-22 directly targets and degrades HMOX1 mRNA in articular chondrocytes and synovial fibroblasts.

### 3.7. Protective Effects of Fucoidan on ACLT-Induced Osteoarthritis in a Rat Model

**Figure 7:**
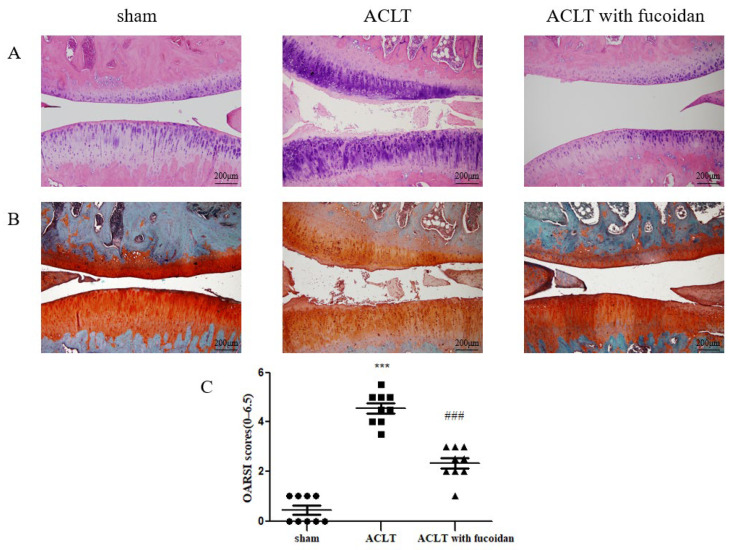
Fucoidan protects against osteoarthritis progression in the anterior cruciate ligament transection (ACLT) rat model (*n* = 9). Joint sections were obtained from each rat group after 12 weeks with or without the oral administration of fucoidan and were visualized using (**A**) H&E staining and (**B**) Safranin O staining to evaluate the thickness of the articular cartilage and loss of glycosaminoglycans (100× magnification). (**C**) Knee joint damage and inflammation were assessed using the Osteoarthritis Research Society International (OARSI) scoring system (scale: 0–6.5) (*** *p* < 0.001 compared with sham; ^###^ *p* < 0.001, ACLT vs. ACLT with fucoidan).

To verify the effect of fucoidan on OA development in vivo, we established an ACLT rat model. The rats were orally administered 100 mg/kg of fucoidan in ddH_2_O or a sham (ddH_2_O alone) once every 2 days for 12 weeks. Histomorphology was assessed using H&E staining (Figure 7A) and Safranin O staining (Figure 7B), and differences between the sham, ACLT, and ACLT with fucoidan groups were evaluated using the Osteoarthritis Research Society International (OARSI) score [26]. Compared to the sham control group, the OARSI score significantly increased to 4.50 ± 0.75-fold (*n* = 9, *p* < 0.001) in the ACLT group. The ACLT group exhibited cartilage denudation, erosion, and proteoglycan loss. Conversely, the ACLT group treated with fucoidan showed a smoother cartilage surface and reduced proteoglycan loss (Figure 7A,B). Furthermore, the OARSI score significantly decreased to 2.33 ± 0.66-fold (*n* = 9, *p* < 0.001) in the fucoidan-treated group compared to in the ACLT group (Figure 7C).

## 4. Discussion

Osteoarthritis is a degenerative disease associated with aging, and it is characterized by a complex pathogenesis that hinders the development of effective disease-modifying therapies. Currently, the primary clinical treatment for OA relies on nonsteroidal anti-inflammatory drugs (NSAIDs), which primarily alleviate symptoms but do not halt disease progression [30]. Given these limitations, there is a growing need for safer and more effective therapeutic alternatives, including nutritional supplements, to support OA management and improve long-term patient outcomes.

Fucoidan, a sulfated polysaccharide derived from brown seaweed, has been widely used as a dietary supplement for health and disease management worldwide. Its primary structure consists of fucose and sulfate, with minor components including xylose, galactose, mannose, and uronic acids [31]. The biological activity of fucoidan is largely influenced by its molecular weight and sulfate content [32,33]. In this study, fucoidan was derived from *Sargassum hemiphyllum* and hydrolyzed using a glycolytic enzyme. The resulting fucoidan, with an average molecular weight of 800 Da, was obtained through filtration using molecular weight cut-off membranes [34]. Fucoidan can modulate key signaling pathways such as PI3K/AKT, MAPK, and apoptosis in cancer therapy [35,36]. Additionally, fucoidan reportedly induces miR-146b-5p expression in mesenchymal stem cells, leading to the inhibition of TRAF6 activation in OA [37]. However, the precise mechanisms by which fucoidan mitigates oxidative stress in articular chondrocytes and synovial fibroblasts remain unclear and require further investigation.

This study aimed to elucidate the therapeutic potential of fucoidan in OA by examining its effects on IL-1β-induced inflammatory cytokines and oxidative stress, key drivers of joint tissue damage in OA. To understand the underlying molecular mechanisms, we investigated fucoidan’s ability to modulate catabolic imbalance. We demonstrated that fucoidan suppressed IL-1β-induced catabolic activity by inhibiting the MAPK and NF-κB pathways while simultaneously activating the Nrf2/HO-1 pathway. This chondroprotective effect was further validated in vitro using a rat animal model. Three months post-surgery, the ACLT-induced OA model showed severe OA and an irregular cartilage surface. In contrast, the ACLT treated with fucoidan group exhibited mild OA and a smooth cartilage surface. Given the potential involvement of microRNAs in these mechanisms, we conducted in silico analyses to identify candidate microRNAs targeting HO-1. Among the five candidate miRNAs screened via q-PCR, miR-22 emerged as a likely regulator. In our cell models, we observed an inverse relationship between miR-22 and HO-1 expression, suggesting that HO-1 could be a direct target of miR-22. This hypothesis was supported by the identification of a seed-complementary sequence to miR-22 within the HO-1 3′UTR using TargetScan. Furthermore, luciferase reporter assays confirmed that miR-22 directly targets HO-1 in joint tissue. Transfection experiments using miR-22 mimics and inhibitors revealed that IL-1β induced an increase in miR-22 expression, while fucoidan treatment conversely downregulated miR-22. This suggests that the protective effects of fucoidan in articular chondrocytes and synovial fibroblasts are, at least in part, mediated through the regulation of miR-22.

Osteoarthritis progression is characterized by joint tissue destruction and inflammation, driven by disruption in the equilibrium between catabolic processes and extracellular matrix synthesis. Chondrocytes play a crucial role in maintaining joint homeostasis and are influenced by various factors, including inflammatory cytokines, matrix metalloproteinases, nitrosative stress, and key signaling pathways [38]. Specifically, inflammatory cytokines such as IL-1β, IL-6, IL-8, and TNF-α contribute to OA pathogenesis [6]. Key enzymes in the breakdown of cartilage in osteoarthritic joints are MMPs, specifically MMP-1, MMP-3, and MMP-13, which are proteolytic enzymes that degrade articular cartilage by cleaving type II collagen [39]. Additionally, nitric oxide, a signaling molecule synthesized from L-arginine by inducible nitric oxide synthase, has been implicated in OA pathogenesis [7]. Furthermore, the MAPK and NF-κB signaling pathways are critical regulators of cellular responses in OA joints, influencing processes such as cell proliferation, differentiation, apoptosis, inflammation, and oxidative stress. ROS, highly reactive molecules, contribute to oxidative stress and joint tissue damage [14]. The presence of nitrite and nitrated products in the ECM indicates that ROS directly impacts cartilage degradation and disrupts ECM homeostasis [14,38].

Nrf2 is a key redox transcription factor capable of inducing the expression of antioxidants, including HO-1 and catalase. HO-1 is a key downstream target of Nrf2 and an inducible isoform enzyme that catalyzes the transformation of heme to carbon monoxide, free iron, and biliverdin [14]. Carbon monoxide is an inhibitor and suppresses the NF-ĸB pathway, which leads to the regulation of pro-inflammatory cytokines in cartilage homeostasis. The induction of Nrf2 and the downstream antioxidant HO-1 is associated with reduced matrix metalloproteinase expression [12,13]. We found that fucoidan inhibited the IL-1β-induced expression of oxidative stress (i-NOS, NO, and ROS) and matrix metalloproteinases (MMP-1 and MMP-3) while increasing the expression of antioxidant proteins (HO-1). These results indicate that the antioxidative effects of fucoidan can be attributed to activation of the Nrf2 pathway. Other molecular mechanisms of fucoidan’s protective effect remain unclear. We propose that the epigenetic regulation of miRNAs may represent the missing piece of the puzzle in OA.

MicroRNAs play a crucial role in OA development by modulating target gene expression [40,41]. Consistent with our findings, miR-22 levels are reportedly elevated in human osteoarthritic cartilage [42] and synovial fluid [17]. The liposomal transfection of miR-22 causes the upregulation of MMP-13 mRNA and the downregulation of ACAN mRNA in normal chondrocytes, while miR-22 neutralization using antisense oligonucleotides significantly reduces MMP-13 expression in osteoarthritic chondrocytes [42]. Furthermore, as per a bioinformatics analysis, miR-22 directly targets the 3′-UTR of BMP-2, demonstrating a negative correlation with BMP-2 expression [17]. These findings highlight the catabolic effects of miR-22 in chondrocytes. In our study, we further confirmed the upregulation of miR-22 in both IL-1β-treated chondrocytes and synovial fibroblasts, and we identified HO-1 mRNA as a direct target of miR-22 in these cells. While IL-1β induced a significant increase in miR-22 expression, fucoidan effectively suppressed this response by downregulating the miR-22 levels in both articular chondrocytes and synovial fibroblasts.

This study presents several notable strengths. Our use of human primary cell cultures enhances the translational relevance of our findings, as these cells are more likely to reflect in vivo conditions. Additionally, the validation of our results using animal models provides further support for the observed effectiveness. However, we acknowledge several limitations. The use of primary cells from different donors introduces potential heterogeneity and donor-specific biases, which could be amplified by the limited passage number (up to passage five). Furthermore, primary cells exhibit a slower growth rate and are generally more challenging to transfect, which might have influenced our experimental design. Due to the random and small sample size of donor cells obtained from total knee replacement surgeries, we were unable to perform a gender-based classification in our analysis. The observed effects remained consistent across donors. A sensitivity analysis showed that the exclusion of any single donor did not materially alter the significance or direction of the results. Finally, it is important to note that the molecular mechanisms identified in this study were verified in vitro only, and further in vivo investigations are warranted to confirm their relevance in a more complex biological system. Future studies could focus on translating these findings into clinical practice by designing and conducting patient-based clinical trials to rigorously evaluate the therapeutic potential of fucoidan in OA. Furthermore, given the established role of upstream epigenetic mechanisms, particularly DNA methylation, in OA pathogenesis, future studies could be directed toward investigating the potential of fucoidan to modulate DNA methylation patterns, thereby serving as a novel therapeutic strategy [43,44].

## 5. Conclusions

In conclusion, this study identified novel fucoidan-related targets of miR-22 in articular chondrocytes and synovial fibroblasts in vitro. By modulating the miR-22/HO-1 pathway, fucoidan reduces oxidative stress and catabolic activity in joint tissues, thereby exerting chondroprotective effects. Notably, our findings provide the first evidence of fucoidan’s epigenetic regulation of miRNAs in IL-1β-induced articular cells. While these results highlight fucoidan’s promising potential as a therapeutic agent for osteoarthritis, further validation in preclinical and clinical models is essential to assess its translational applicability in managing the disease.

## Data Availability

The original contributions presented in this study are included in the article. Further inquiries can be directed to the corresponding author.
